# Influence of chronic kidney disease and other risk factors pre-heart transplantation on malignancy incidence post-heart transplantation

**DOI:** 10.3389/fcvm.2023.1145996

**Published:** 2023-04-03

**Authors:** Stefan Roest, Muhammed T. Gürgöze, Wida S. Cherikh, Josef Stehlik, Eric H. Boersma, Felix Zijlstra, Olivier C. Manintveld

**Affiliations:** ^1^ Department of Cardiology, Thorax Center, Erasmus MC, University Medical Center Rotterdam, Rotterdam, Netherlands; ^2^ Erasmus MC Transplant Institute, Erasmus MC, University Medical Center Rotterdam, Rotterdam, Netherlands; ^3^ United Network for Organ Sharing, Richmond, VA, United States; ^4^ Division of Cardiovascular Medicine, U.T.A.H. Cardiac Transplant Program, University of Utah Health Salt Lake City, Utah, UT, United States

**Keywords:** cancer, heart transplantation, malignancies, chronic kidney disease, death-adjusted incidence

## Abstract

**Aims:**

Chronic kidney disease (CKD) pre-heart transplantation (HTx) has been proposed as a risk factor for malignancy risk post-HTx. Using multicenter registry data, our aim was to calculate the death-adjusted annual incidence of malignancies post-HTx, corroborate the association between CKD pre-HTx and malignancy risk post-HTx, and determine other risk factors for post-HTx malignancies.

**Methods and materials:**

We used data from patients transplanted in North American HTx centers between January 2000 and June 2017 and registered in the International Society for Heart and Lung Transplantation Thoracic Organ Transplant Registry. We excluded recipients with missing data on post-HTx malignancies, heterotopic heart transplant, retransplantation, multi-organ transplantation, and patients with a total artificial heart pre-HTx.

**Results:**

Overall, 34,873 patients were included to determine the annual incidence of malignancies, 33,345 patients were included in the risk analyses. The incidence of any malignancy, solid-organ malignancy, post-transplant lymphoproliferative disease (PTLD), and skin cancer adjusted for death 15 years post-HTx, was 26.6%, 10.9%, 3.6%, and 15.8% respectively. Besides widely acknowledged risk factors, CKD stage ≥4 pre-HTx was associated with the development of all malignancies post-HTx (HR 1.17 compared to CKD stage 1, *p* = 0.023), as well as solid-organ malignancies (HR 1.35, *p* = 0.01), but not for PTLD (HR 0.73, *p* = 0.057), and skin cancer (HR 1.06, *p* = 0.59).

**Conclusion:**

Risk of malignancy post-HTx remains high. CKD stages ≥4 pre-HTx was associated with an increased risk for any malignancy and solid-organ malignancy post-HTx. Strategies to mitigate the impact of pre-HTx patient factors on the risk of post-HTx malignancy are needed.

## Graphical Abstract

Influence of Chronic Kidney Disease and Other Risk Factors Pre-Heart Transplantation on Malignancy Incidence Post-Heart Transplantation Stefan Roest, Muhammed T. Gürgöze, Wida S. Cherikh, Josef Stephlik, Eric H. Boersma, Felix Zijlstra, Olivier C. Manintveld* in the largest international registry for heart transplant recipients (UNOS database), the influence of chronic kidney disease and other risk factors for malignancies post-heart transplantation was investigated. In this study it is demonstrated that the incidence of malignancies post-transplantation is high, with an eFGR < 30 ml/min/1.73 m^2^ as a significant risk factor for any malignancy and solid-organ malignancies. Further studies investigating strategies to mitigate the impact of pre-HTx patient factors on the risk of post-HTx malignancy are needed.

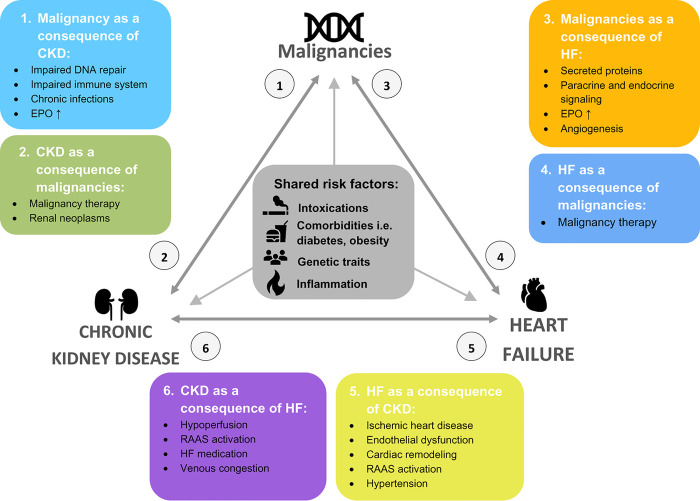

## Introduction

Several epidemiologic studies have demonstrated that patients with heart failure have an increased risk for malignancy development (1, 2), while others found no association (3). The idea that there is an association between heart failure and malignancy development was strengthened by a study by Meijers et al. (4). In that study, mice who developed heart failure after a myocardial infarction, had an increased risk of malignancy development (4). One of the circulating factors responsible for this was found to be serine proteinase inhibitor A3 (SerpinA3) (4). The authors postulated the hypothesis that heart failure leads to the secretion of proteins by the myocardium inducing tumor growth (4). In contrast, a study by Koelwyn et al. demonstrated that myocardial infarction induced immune reprogramming which led to an increased breast cancer tumor growth (5). Interestingly, others found an association between cardiovascular risk factors and malignancies instead of heart failure itself (6).

A common comorbidity in heart failure patients is chronic kidney disease (CKD) (7). Several studies have shown that patients with an impaired kidney function have an impaired immune system (one of the pathways *via* uremia) (8, 9). Furthermore, it is known that patients post-heart transplantation (HTx) have an increased risk for malignancies due to the extended immunosuppressive therapy (10). However, the exact risk patients post-HTx have is unknown since no studies have corrected for death as a competing outcome. In light of these studies, we have previously performed a single-center study hypothesizing that heart failure duration and CKD increased the risk of malignancy post-HTx (11). We demonstrated that CKD pre-HTx, and not heart failure duration, significantly increased the risk for malignancy post-HTx, concluding that the relationship between heart failure and malignancy could be influenced by CKD (11). In the current study, the annual cumulative incidence of malignancies post-HTx with death as a competing endpoint was investigated. Additionally, we investigated whether the association between CKD and malignancy development could be confirmed in a large multicenter heart transplantation registry. Finally, other risk factors for malignancy post-HTx were examined.

## Materials and methods

### Study population

In this retrospective cohort study, data routinely collected in the International Society for Heart and Lung Transplantation (ISHLT) Thoracic Organ Transplant (TTX) Registry was analyzed. Since data on malignancy are more consistently available from the North American centers, only data from these centers were included.

We included recipients who underwent primary, orthotopic, heart-only transplants between January 1, 2000 and June 30, 2017. We excluded recipients with missing data on post-HTx malignancies, heterotopic heart transplants, retransplantations, multi-organ transplantations, and patients with a total artificial heart (TAH) pre-HTx. We used two cohorts of patients for our analyses. First, a cohort of patients who survived 90 days after transplant for estimation of the incidence rates of malignancies post-HTx in the presence of death. Second, a cohort of patients who survived past 1 year after HTx was used to perform the multivariable Cox regression models. This was done since we included rejection at 1 year after HTx as one of the covariates.

Serum creatinine pre-HTx was defined as the most recent creatinine prior to transplantation. Recipient CKD stage pre-HTx was calculated using the Cockcroft-Gault eGFR formula (12), since race is not collected in the database. Accordingly, patients were classified by CKD stage as defined by the KDIGO (13) from stage 1 to 5. Patients were divided into three groups based on the heart failure etiology: ischemic cardiomyopathy, non-ischemic cardiomyopathy and other. The latter group included a wide range of diagnoses such as congenital heart disease, restrictive cardiomyopathy, valvular cardiomyopathy, and arrhythmogenic right ventricular cardiomyopathy. Death events described in the manuscript reflect all-cause deaths.

This study is in accordance with the declaration of Helsinki and was approved by the Medical Ethical Review Committee (MEC-2021-731).

### Statistical analysis

To account for death as a competing outcome in the analysis to estimate the annual cumulative incidence of malignancy post-HTx, a competing risk extension of the Kaplan-Meier method was performed. This was divided into three groups: any malignancy, solid-organ malignancy and PTLD.

To investigate the risk of CKD pre-HTx on malignancy risk post-HTx, four multivariable Cox regression models were created. The endpoint of these four multivariable Cox regression models were: any malignancy, solid-organ malignancy, post-transplant lymphoproliferative disease (PTLD) and skin malignancy. In the solid-organ malignancy analysis both skin cancers as well as PTLD were excluded. A full list of covariates explored on these analyses is included in Supplementary Table S1. Covariates were not included in the Cox models when data were missing in >50% of the included recipients. If <50% of values were missing, data were imputed using a multiple imputation method. In order to allow for the most flexible fit of the functional form, continuous risk factors were modeled as restricted cubic spline with three knots. The median of a continuous variable was used as the reference group. For categorical variables, the category with the highest frequency was used as the reference group. An iterative backward method was used for variable selection. In order to determine the risk factors to retain in the final model, only variables with overall *p* < 0.10 were included in the final model. Since recipient CKD stage pre-HTx was of primary interest in this study, this variable was retained in all models regardless of the statistical significance. Given the small number of patients in CKD stage 4 and 5, these were combined into one group.

A *p*-value <0.05 was considered statistically significant. Statistical analyses were performed using SAS v9.4 (SAS Institute, Inc., Cary, NC), IBM SPSS statistics 25 (IBM Corp., New York, United States) and R Version 4.0.2 (R: A language and environment for statistical computing. R Foundation for Statistical Computing, Vienna, Austria. https://www.R-project.org/).

## Results

### Study population

A flow diagram of the study population is demonstrated in Figure S1. Between HTx and 90 days post-transplant 3,408 patients died, while 5,724 patients died between HTx and 1 year post-transplant. A total of 39,635 pediatric and adult HTx recipients survived at least 90 days post-HTx. Of these, 4,762 were excluded from further analysis due to the following reasons (some patients excluded based on several reasons): missing malignancy data post-HTx (*n* = 2,057), heterotopic heart transplant (*n* = 33), retransplantation (*n* = 1,411), multi-organ transplant (*n* = 1,347), pre-HTx TAH (*n* = 310). Therefore, for the analysis of malignancy incidence, 34,873 recipients were included in the analysis of any malignancy outcome, 34,852 for solid-organ malignancy outcome, 34,202 for PTLD outcome and 34,265 for skin malignancy outcome.

A total of 37,319 pediatric and adult HTx recipients survived past 1 year of HTx. Of these, 3,974 were excluded after applying the exclusion criteria. The reasons included (some patients were excluded based on several reasons): missing malignancy data post-HTx (*n* = 1,431), recipients of heterotopic heart transplant (*n* = 26), retransplantation (*n* = 1,302), recipients of multi-organ transplant (*n* = 1,250) and recipients with a TAH pre-HTx (*n* = 285). For the Cox regression analyses, 33,345 recipients were included for any malignancy outcome, 33,324 for solid-organ malignancy outcome, 32,678 for PTLD outcome, and 32,738 for skin malignancy outcome.

The baseline characteristics of the patients are shown in Table 1. Data on missingness is shown in Supplementary Table S2. Overall, the minority of recipients were female (28%) and the median age was 53 [interquartile range (IQR) 36–61] years. Ischemic cardiomyopathy was the reason for HTx in 35% of the patients. Median estimated glomerular filtration rate (eGFR) was 77 [IQR 57–103] with a distribution over the CKD stages of 36%, 35%, 15%, 8%, and 5% for CKD stage 1, 2, 3A, 3B and ≥4, respectively. The median survival [IQR] of patients surviving more than 90 days post-HTx was 6.0 [3.0–10.0] years, while patients surviving more than 1 year post-HTx had a median survival of 6.1 [3.3–10.1] years.

**Table 1:** Baseline characteristics of patients included in the study.

	Survival ≥90 days post-HT (*n* = 34,873)	Survival ≥1 year post-HT (*n* = 33,345)
**Donor**		
Age (years)	26 [19–39]	26 [19–39]
Female	10,710 (31)	10,249 (31)
History of cancer	477 (1)	451 (1)
Donor CMV positive	20,654 (59)	19,709 (59)
Donor EBV positive	20,857 (60)	19,951 (60)
**Recipient**		
Age at HTx (years)	53 [36–61]	53 [36–61]
Female	9,737 (28)	9,296 (28)
**Transplant era**		
2000–2008	16,033 (46)	15,333 (46)
2009–June 2017	18,840 (54)	18,012 (54)
Ischemic time (hours)	3.2 [2.5–3.9]	3.2 [2.5–3.9]
**Etiology heart failure**		
Ischemic CMP	12,105 (35)	11,592 (35)
Non-ischemic CMP	14,281 (41)	13,656 (41)
Other	8,487 (24)	8,097 (24)
CMV positive serostatus	18,986 (54)	18,142 (54)
EBV positive serostatus	23,560 (68)	22,515 (68)
VAD pre-HTx	9,944 (29)	9,440 (28)
Malignancies pre-HTx	2,215 (6)	2,131 (6)
Diabetes pre-HTx	7,410 (21)	7,060 (21)
Hypertension pre-HTx	11,559 (33)	11,071 (33)
Smoking pre-HTx	10,728 (31)	10,259 (31)
Creatinine pre-HTx (Mg/dl)	1.1 [0.8–1.4]	1.1 [0.8–1.4]
eGFR pre-HTx (ml/min/1.73 m^2^)	77 [57–103]	77 [57–103]
**KDIGO CKD stages**		
CKD stage 1	12,423 (36)	11,913 (36)
CKD stage 2	12,238 (35)	11,749 (35)
CKD stage 3A	5,149 (15)	4,908 (15)
CKD stage 3B	2,623 (8)	2,487 (8)
CKD stage 4/5	1,713 (5)	1,603 (5)
**Induction therapy**		
No induction	16,319 (47)	15,629 (47)
IL-2RA	8,929 (26)	8,532 (26)
Monoclonal	1,032 (3)	1,001 (3)
Polyclonal	7,699 (22)	7,336 (22)
Multiple induction types	584 (2)	553 (2)
Acute rejection until discharge	2,945 (8)	2,782 (8)
Acute rejection discharge and 1 year	6,659 (19)	6,150 (18)
Follow-up duration (years)	5.9 [3.0–10.0]	6.1 [3.3–10.1]

Baseline characteristics of the patients included in the study. Categorical variables are demonstrated with absolute numbers (percentages). Continuous variables are demonstrated with medians [25th–75th percentile (interquartile range)]. Abbreviations: CKD, chronic kidney disease; CMP, cardiomyopathy; CMV, cytomegalovirus; EBV, Epstein Barr virus; eGFR, estimated glomerular filtration rate; HTx, heart transplant; IL-2RA, interleukin-2 receptor antagonists (e.g. basiliximab, daclizumab); KDIGO, Kidney Disease: Improving Global Outcomes; VAD, ventricular assist device.

### Rate of malignancy post-heart transplant

**Figure 1:**
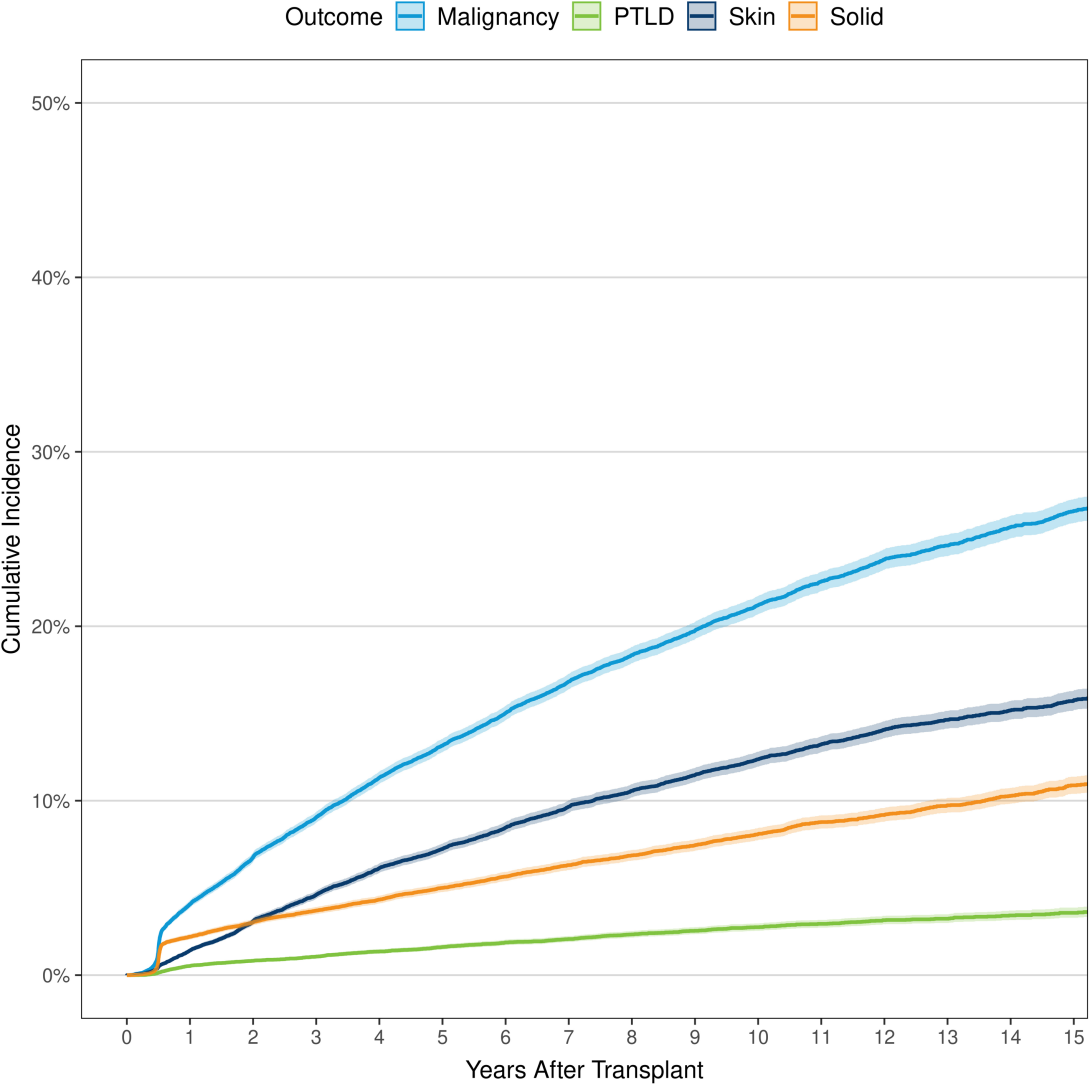
Cumulative incidence of any malignancy, solid cancer, post-transplant lymphoproliferative disease, and skin cancer for recipients of primary, orthotopic heart transplants between January 1, 2000 and June 30, 2017 in North America. PTLD, post-transplant lymphoproliferative disease.

Figure 1 and Supplementary Table S3 demonstrate the annual cumulative incidence of any malignancy, solid-organ malignancy, PTLD, and skin cancer with death as a competing risk outcome. By 10 years post-HTx, the incidence was 21.2% for any malignancy, 8.1% for solid-organ malignancy, 2.8% for PTLD, and 12.4% for skin cancer. These rates increased to 26.6% for any malignancy, 10.9% for solid-organ malignancy, 3.6% for PTLD, and 15.8% for skin cancer by 15 years post-HTx.

### Risk factors for post-transplant malignancy

#### Any malignancy

**Figure 2:**
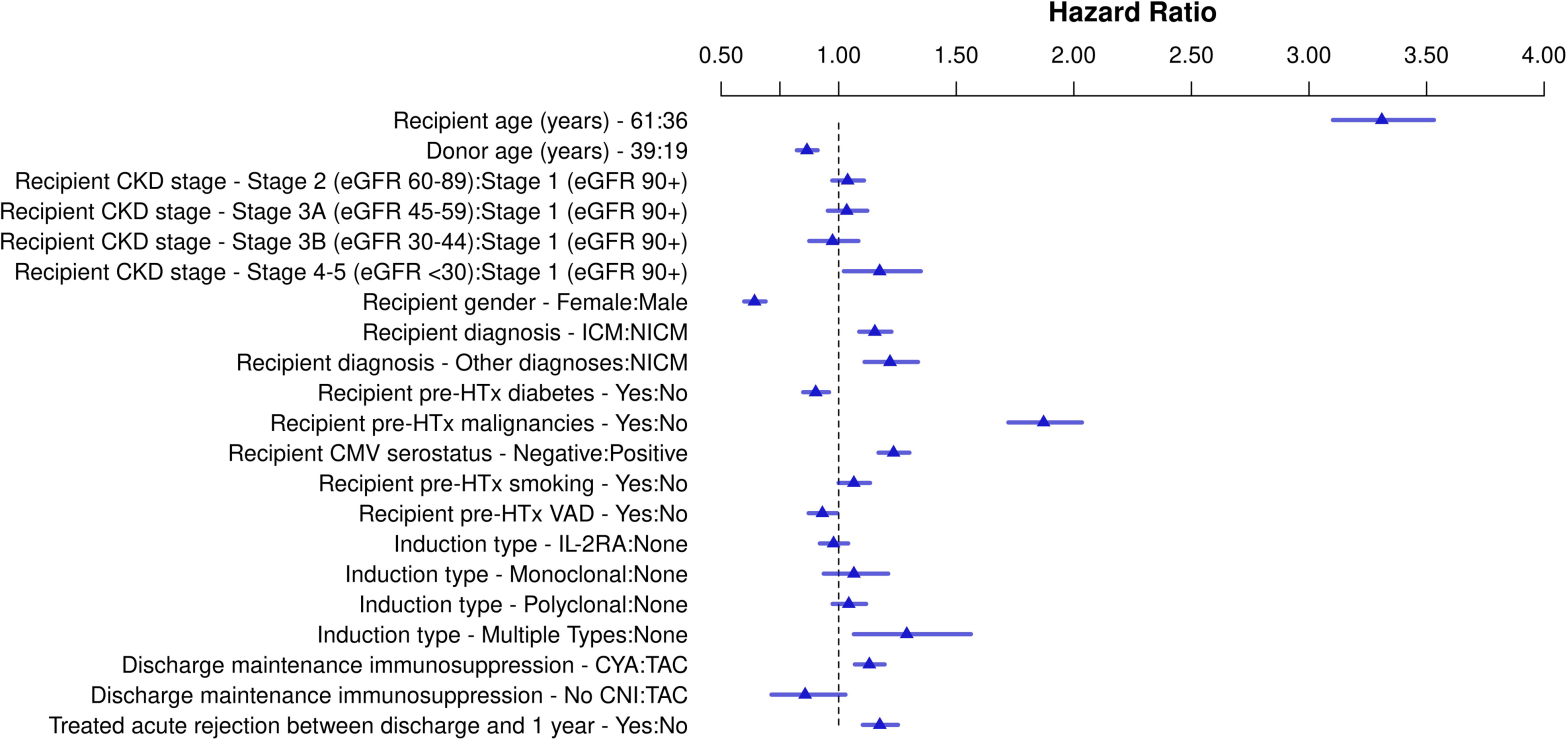
Schematic overview for risk factors for all malignancy post-heart transplant. Hazard ratios for continuous variables are shown for the third quartile vs. the first quartile. CKD, chronic kidney disease; CNI, calcineurin inhibitor; CMV, cytomegalovirus; CYA, cyclosporine; eGFR, estimate glomerular filtration rate; HTx, heart transplantation; ICM, ischemic cardiomyopathy; IL-2RA, interleukin-2 receptor antagonist; NICM, non-ischemic cardiomyopathy; TAC, tacrolimus; VAD, ventricular assist device.

Of the 33,345 HTx recipients included in the study, 6,093 (18.3%) developed a malignancy post-HTx during follow-up. The results of the Cox Regression analysis examining risk factors for any malignancy post-HTx are shown in Figure 2, Supplementary Table S4, and Figure S2. Recipient CKD stage ≥4 pre-HTX was independently associated with an increased risk for malignancy development post-HTx compared to recipients with CKD stage 1 (HR 1.17, *p* = 0.023). CKD stages 2, 3A and 3B were not associated with an increased risk. Other risk factors for an increased risk of malignancy development post-HTx included male sex (HR 1.56, *p* < 0.001), older recipient age (*p* < 0.001), younger donor age (*p* = 0.01), ischemic cardiomyopathy (HR 1.15, *p* < 0.001) and other diagnoses of HF etiology (HR 1.22, *p* < 0.001) compared to non-ischemic cardiomyopathy, recipient history of malignancy pre-HTx (HR 1.87, *p* < 0.001), recipient CMV negative serostatus (HR 1.23, *p* < 0.001), multiple types of induction (HR 1.29, *p* = 0.009), cyclosporine as maintenance immunosuppression at discharge (compared to tacrolimus) (HR 1.13, *p* < 0.001), and treatment for acute rejection between discharge and 1 year (HR 1.17, *p* < 0.001). Female recipients had lower risk of malignancy after transplant compared to male recipients (HR 0.64, *p* < 0.001), recipient pre-HTx diabetes (HR 0.90, *p* = 0.001) and ventricular assist device (VAD) used pre-HTx (HR 0.93, *p* = 0.03) were associated with a reduced risk to develop any malignancy post-HTx.

#### Solid-organ malignancy

**Figure 3:**
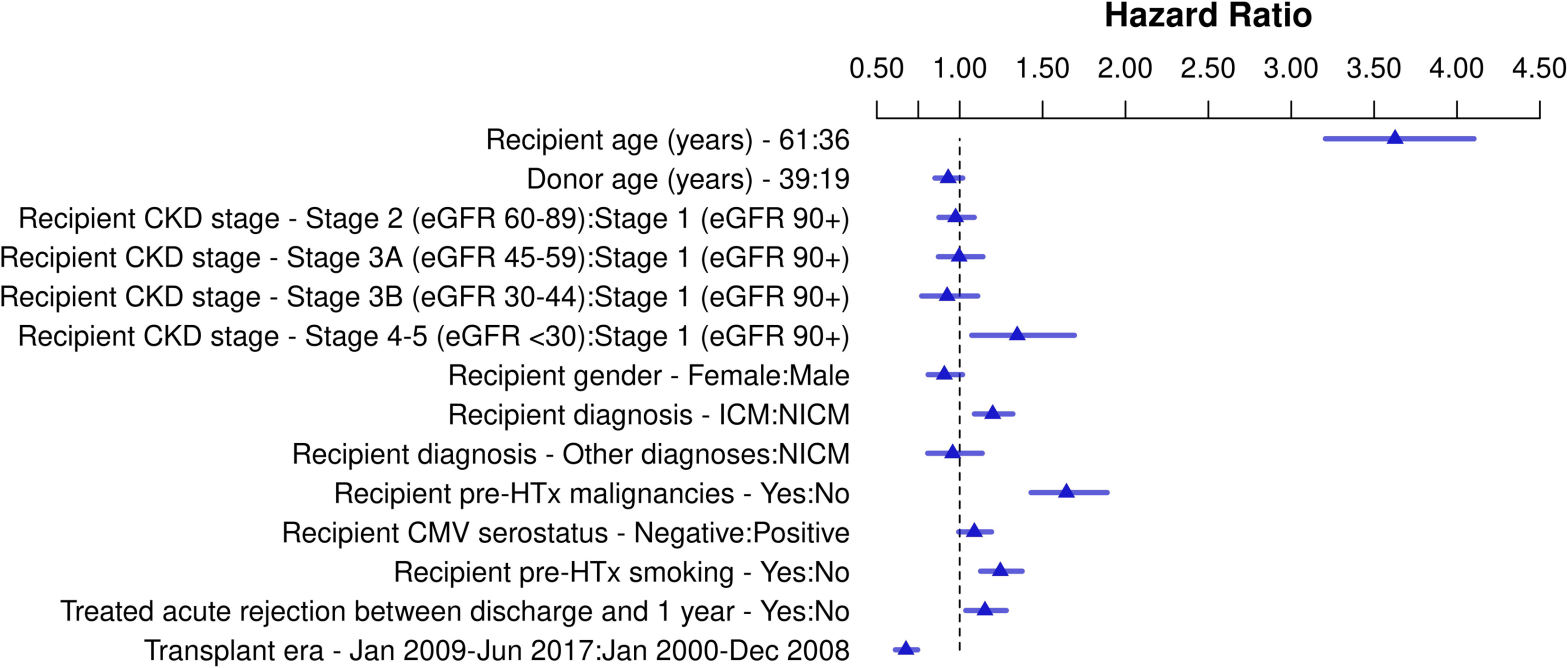
Schematic overview for risk factors for solid-organ malignancy post-heart transplant. Hazard ratios for continuous variables are shown for the third quartile vs. the first quartile. CKD, chronic kidney disease; CMV, cytomegalovirus; eGFR, estimated glomerular filtration rate; HTx, heart transplantation ICM, ischemic cardiomyopathy, NICM, non-ischemic cardiomyopathy.

Of the 33,324 HTx recipients included in the solid-organ malignancy analysis, 2,415 (7.3%) developed a solid-organ malignancy post-HTx. In Figure 3, Supplementary Table S5, and Figure S3, the results of the multivariable Cox regression are shown. Recipient CKD stage ≥4 pre-HTx was also significantly associated with solid-organ malignancies post-HTx (HR 1.35, *p* = 0.01) compared to recipients with CKD stage 1. Other risk factors for solid-organ malignancies included older recipient age (*p* < 0.001), ischemic cardiomyopathy (HR 1.20, *p* < 0.001), recipient history of malignancy pre-HTx (HR 1.64, *p* < 0.001), recipient history of smoking (HR 1.25, *p* < 0.001), and treatment for acute rejection between discharge and 1 year after transplant (HR 1.15, *p* = 0.009). Solid-organ malignancy risk was lower in recipients who were transplanted in the recent era (HR 0.68, *p* < 0.001).

#### Post-transplant lymphoproliferative disease

**Figure 4:**
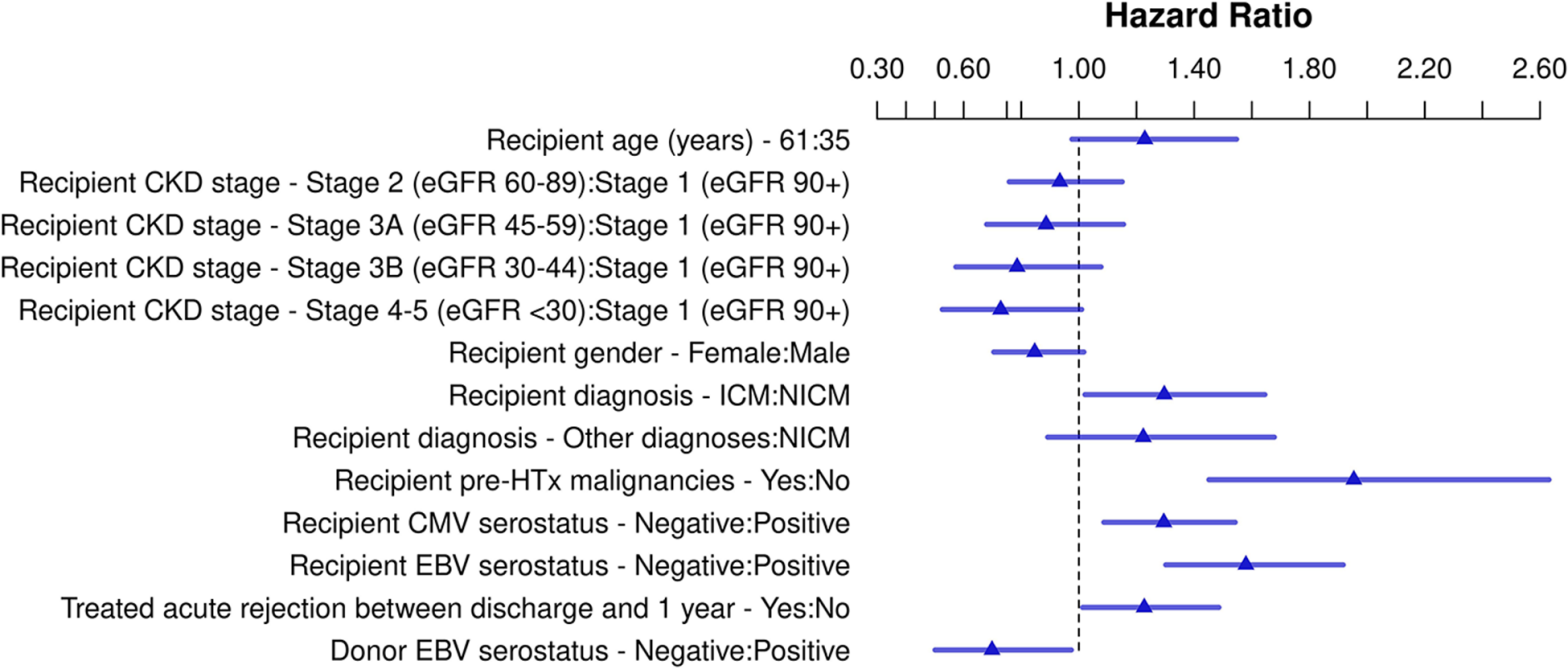
Schematic overview for risk factors for post-transplant lymphoproliferative disease post-heart transplant. Hazard ratios for continuous variables are shown for the third quartile vs. the first quartile. Abbreviations: CKD, chronic kidney disease; CMV, cytomegalovirus; EBV, Epstein Barr virus; eGFR, estimated glomerular filtration rate; HTx, heart transplantation; ICM, ischemic cardiomyopathy; NICM, non-ischemic cardiomyopathy.

Of the 32,678 HTx recipients included in the PTLD analysis, 760 (2.3%) developed PTLD post-HTx. The results of the multivariable Cox regression are shown in Figure 4, Supplementary Table S6, and Figure S4. Recipient CKD stage ≥4 was not associated with an increased risk of PTLD compared to patients with CKD stage 1 (HR 0.73, *p* = 0.057). Ischemic cardiomyopathy (HR 1.20, *p* = 0.033), recipient history of malignancy pre-HTx (HR 1.95, *p* < 0.001), recipient CMV negative serostatus (HR 1.29, *p* = 0.004), recipient EBV negative serostatus (HR 1.58, *p* < 0.001), and treatment for acute rejection between discharge and 1 year post-transplant (HR 1.23, *p* = 0.036) were associated with increased risk for PTLD. Additionally, donor EBV negative serostatus was associated with decreased risk of PTLD (HR 0.70, *p* = 0.034). Recipient age was associated with PTLD—the risk was markedly increased in pediatric transplant recipients, and in recipients >60 years of age at transplant (Figure S4, *p* < 0.001).

#### Skin malignancies

**Figure 5:**
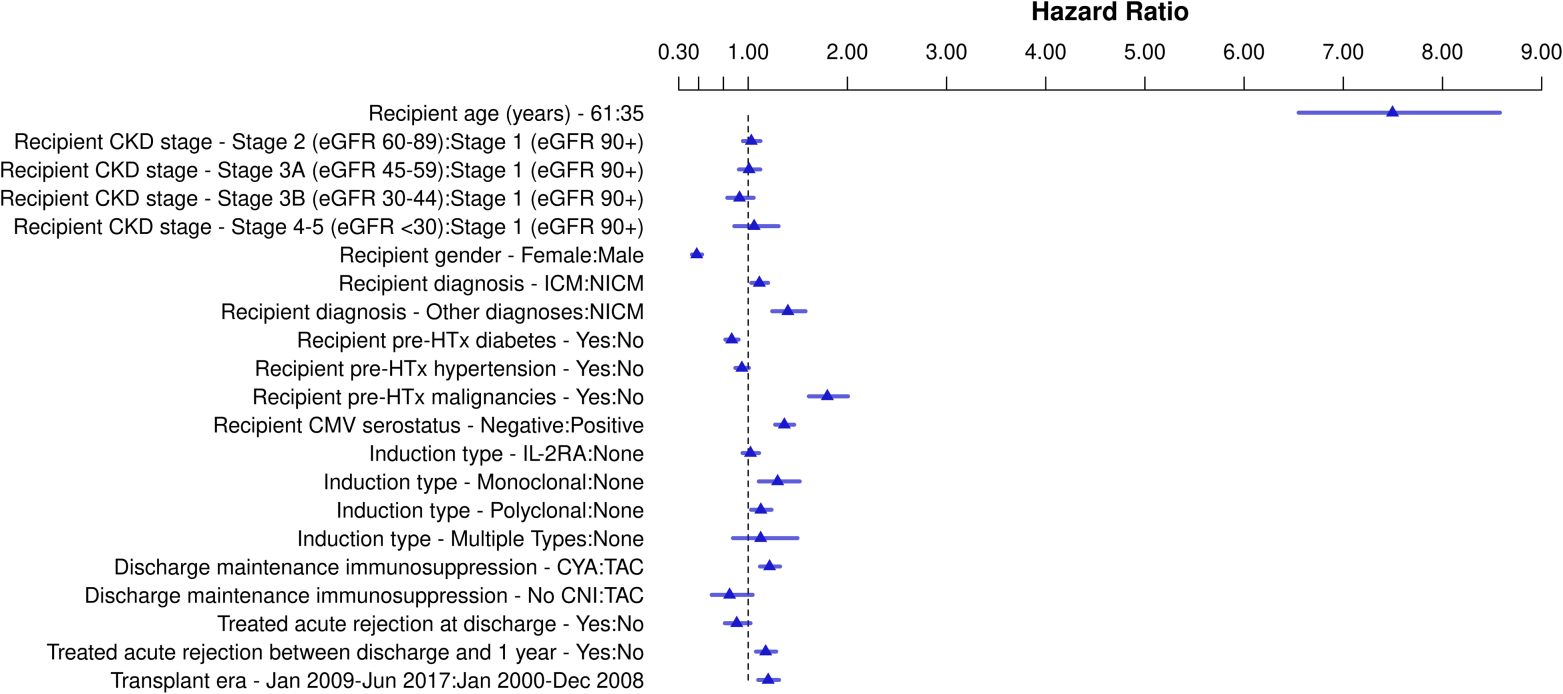
Schematic overview of risk factors for skin malignancies post-heart transplant. Hazard ratios for continuous variables are shown for the third quartile vs. the first quartile. Abbreviations: CKD, chronic kidney disease; CMV, cytomegalovirus; CYA, cyclosporine; eGFR, estimated glomerular filtration rate; HTx, heart transplantation; ICM, ischemic cardiomyopathy; IL-2RA, interleukin-2 receptor antagonist; NICM, non-ischemic cardiomyopathy; TAC, tacrolimus.

Of the 32,738 transplants included in the skin cancer analysis, 3,439 (10.5%) developed a skin malignancy post-HTx. The results of the multivariable Cox regression are shown in Figure 5, Supplementary Table S7, and Figure S5. Recipient CKD stage ≥4 was not associated with an increased risk for skin malignancies compared to patients with CKD stage 1 (HR 1.06, *p* = 0.59). Other risk factors for skin malignancies include: older recipient age (*p* < 0.001), ischemic cardiomyopathy (HR 1.11, *p* = 0.006), malignancies pre-HTx (HR 1.80, *p* < 0.001), recipient negative CMV serostatus (HR 1.36, *p* < 0.001), recipient with monoclonal (HR 1.30, *p* = 0.001) or polyclonal induction (HR 1.13, *p* = 0.01), recipient discharged on CYA compared to TAC (HR 1.22, *p* < 0.001), and treatment for acute rejection between discharge and 1 year (HR 1.18, *p* < 0.001). Female recipients (HR 0.48, *p* < 0.001) and recipients with pre-HTx diabetes (HR 0.83, *p* < 0.001) significantly reduced risk for skin malignancies post-HTx.

## Discussion

The results of our study illustrate that the rate of malignancy after transplant remains high. The rate of any malignancy, solid-organ malignancy, PTLD and skin malignancy at 15 years after transplant, after adjustment for competing risk of death, was 26.6%, 10.9%, 3.6%, and 15.8%, respectively. In addition to patient characteristics widely acknowledged to represent risk for post-transplant malignancy, we also show that CKD stage 4 and 5 (eGFR <30 ml/min/1.73 m^2^) pre-HTx is a significant risk factor for the development of any malignancy and solid-organ malignancy post-HTx. CKD was not associated with development of PTLD and skin malignancies.

Recently, there has been growing evidence that cardiovascular diseases such as myocardial infarctions and heart failure can induce malignancies as well (1, 2). This has led to the introduction of cardio-oncology pathways demonstrating the possible correlation between heart failure and malignancies (14) and a roadmap by the Heart Failure Association of the European Society of Cardiology in order to streamline the investigations to these pathways (15). We previously have performed a single-center study in HTx recipients, which demonstrated that chronic kidney disease increased the risk for malignancies post-HTx (11). Due to the sample size, we were not able to classify the kidney function according to the CKD stages, or to test whether patients with an ischemic cardiomyopathy pre-HTx had an increased risk for malignancies post-HTx (11). In the current multicenter study, we were able to include CKD stages as well as an analysis of the cardiomyopathy type pre-HTx, and have shown that patients with an eGFR <30 ml/min/1.73 m^2^ have an increased risk of any malignancy and solid-organ malignancy post-HTx. These results extend the findings of studies that demonstrated higher inflammatory state and weakened immune response in patients with eGFR <30 ml/min/1.73 m^2^ (8, 9). This supports our hypothesis that heart failure, chronic kidney disease and malignancies are interconnected (Figure S6, Graphical abstract) (11). Each disease can result in the occurrence of another comorbidity in this figure, while they share similar risk factors such as hypertension, smoking, obesity and diabetes mellitus (11, 16, 17). Furthermore, genetic traits and inflammation play a role in all three diseases (11, 16, 17). Our analysis did not explore combined heart-kidney transplantation. Whether concurrent kidney transplant in heart transplant recipients with renal dysfunction may abrogate the risk of cancer after transplant is not known. While kidney transplant results in improved GFR, only one kidney is transplanted and the recipient's failing kidneys remain *in situ*.

In our analysis, ischemic cardiomyopathy increased the risk for malignancies post-HTx in all subgroup analyses. This is in line with cohort studies demonstrating that patients after a myocardial infarction have an increased malignancy risk, with an incidence rate ratio of 1.24 in the study of Banke et al. and a hazard ratio of 1.71 after adjustment in the study by Hasin et al. (1, 2). Furthermore, this confirms the findings by Meijers et al. who used an animal model with mice after myocardial infarction who did or did not develop heart failure afterwards (4). This could be explained by the circulating factors demonstrated in the same study such as SerpinA3 (4). Patients with increased cardiac markers had a hazard ratio of 1.06–1.10 for the onset of new-onset cancer as well. While, another study found that immune reprogramming occurs after myocardial infarction increasing the risk for breast cancer (5). In this study, patients had a 59% increased risk of recurrence of breast cancer after incident cardiovascular event compared to patients without cardiovascular event (5). Large prospective cohort studies are needed to definitively elucidate the association between heart failure and the development of malignancies during follow-up including important risk factors, biomarkers, and analysis of the immune response/immune reprogramming.

Several other risk factors were demonstrated in the current study that are already known in literature. For example, it is well-known that the recipient age is an important factor post-HTx (11, 18–20). Furthermore, female recipients are less likely to develop malignancies post-HTx which is conform what is seen in the general population and other HTx studies (18–22). This is could be due to the hormonal influences which decreases the risk for malignancies. Induction therapy is often used at the time of the HTx (23). In our single-center study, we could not exclude that a combination of induction therapy, CKD and heart failure was the cause of malignancy risk post-HTx (11). However, in the current study, we have seen that only the use of multiple induction types pre-HTx, increased the risk for malignancies post-HTx. In our opinion, this should thus be avoided whenever possible. A multicenter registry study from Spain has suggested that induction therapy could increase malignancy risk post-HTx (18, 24). This risk was reduced when patients received antiviral drugs in the first 3 months post-HTx (18). However, this study was, for the most part, performed before the introduction of modern immunosuppressive therapy such as tacrolimus and mycophenolate mofetil. An unexpected finding of our study was that ventricular assist device (VAD) use was associated with a lower risk of all malignancies. This could possibly be explained by the improvement of hemodynamics, subsequent improvement of kidney function and a reduced inflammatory response. However, residual confounding might also explain these findings. Lastly, in our study, diabetes mellitus pre-HTx was associated with reduced risk of all malignancies post-HTx. This is somewhat contradictory, given the fact that diabetes has been described as a risk factor for malignancy development (25). Recent investigations suggested that metformin reduces the incidence and mortality due to malignancies (26). This could have contributed to our findings. Residual confounding might also explain these findings. The question remains how the impact of CKD pre-HTx on malignancy development post-HTx should be mitigated. Some strategies could include durable VAD support in selected patients with cardiorenal syndrome aiming for improved kidney function, combined heart-kidney transplantation in patients with more advanced forms of renal dysfunction or renal-sparing immunosuppressive approaches post-HTx in at risk patients.

This study has some limitations. In the ISHLT registry, no information is gathered on the race of the patient. This has two disadvantages; first it is not possible to use the Chronic Kidney Disease Epidemiology Collaboration (CKD-EPI) formula, which is most commonly used today. Instead we used the Cockcroft-Gault eGFR formula. This could lead to the underestimation of the eGFR in some patients. Secondly, we were not able to compare differences between ethnicities both on risk factors as well as malignancy incidence. Furthermore, serum creatinine value obtained closest to transplant was available in the Registry. It is possible, however, that creatinine fluctuated in some transplant candidates in the days and weeks prior to transplant. Another limitation of our study is that only patients transplanted in North America were included, since data on malignancy incidence were more consistently available from centers in North America. This could limit the generalizability of our results to other regions. We included patients of all ages in the analysis. While this could limit the generalizability of the results to populations with only pediatric or adult recipients this should have been mitigated by adjusting for recipient age. We used Cox multivariable modeling to identify factors independently associated with outcomes of interest, but we acknowledge that residual confounding may be present.

In conclusion, despite decrease in post-transplant malignancy in recent era, its incidence remain high. We found that CKD stage 4 and 5 (eGFR <30 min/ml/1.73 m^2^) pre-HTx was a significant risk factor for the development for any malignancy and solid-organ malignancy, but not for PTLD, and skin malignancies. Further investigations are needed in order to determine how to best mitigate the risk of CKD on development of malignancy.

## Data Availability

The data of this study are not openly available but are available from the corresponding author on reasonable request.
