# Supplementary material for: Influence of chronic kidney disease and other risk factors pre-heart transplantation on malignancy incidence post-heart transplantation
Source: Front Cardiovasc Med. 2023 Apr 3;10:1145996. doi: 10.3389/fcvm.2023.1145996 (PMC10106779; doi:10.3389/fcvm.2023.1145996)
Supplement: Supplementary file 1 [file Datasheet1.docx]

**Supplemental Material**

**Supplemental Table S1. Overview of variables included into the univariable Cox regression analysis.**

| **Variable** |
| --- |
| Recipient age at HTx (years) (continuous) |
| Recipient gender (male, female) |
| Recipient indication for heart transplant (diagnosis):   - Non-ischemic cardiomyopathy (NICM) - Ischemic cardiomyopathy (ICM) - Other |
| Recipient CKD stage as estimated by the Cockcroft-Gault eGFR formula:   - CrCl (male): ([140-age] × weight in kg)/(serum creatinine x 72); - CrCl (female) = CrCl (male) × 0.85;   and was categorized as:   - Stage 1: GFR ≥90 - Stage 2: GFR 60-89 - Stage 3A: GFR 45-59 - Stage 3B: GFR 30-44 - Stage ≥4-5: GFR <30 |
| Recipient pre-HTx diabetes (no, yes) |
| Recipient pre-HTx diabetes (no, yes) |
| Recipient pre-HTx hypertension (no, yes) |
| Recipient pre-HTx malignancies (no, yes) |
| Recipient CMV serostatus (positive, negative) |
| Recipient EBV serostatus (positive, negative) |
| Recipient pre-HTx smoking (no, yes) |
| Recipient pre-HTx VAD (no, yes) |
| Recipient induction therapy:   - None - Monoclonal - Polyclonal - IL-2R |
| Recipient maintenance immunosuppression at discharge   - Tacrolimus - Cyclosporine - None/other |
| Recipient treated acute rejection at discharge (no, yes) |
| Recipient treated acute rejection between discharge and one year (no, yes) |
| Donor age (years) (continuous) |
| Donor gender (male, female) |
| Donor EBV serostatus (positive, negative) |
| Donor history of cancer (no, yes) |
| Ischemic time (hours) (continuous) |
| Transplant era (2000-2008, 2009-June 2017) |

Overview of variables included into the univariable Cox regression analysis. When a p-value was < 0.10, the variable was included in the final multivariable Cox regression analysis.

Abbreviations: CrCl, serum creatinine; CMV, cytomegalovirus; EBV, Epstein Barr Virus; GFR, glomerular filtration rate; HTx, heart transplantation; IL-2R, interleukin-2 receptor blocker (i.e. basiliximab); VAD, ventricular assist device.

**Supplemental Table S2. Missing values baseline characteristics of patients included in the study**

|  | Survival ≥ 90 days  (n=34,873)  N (%) missing | Survival ≥ one year  (n=33,345)  N (%) missing |
| --- | --- | --- |
| **Donor** |  |  |
| Age (years) | 3 (0) | 3 (0) |
| Gender | 2 (0) | 2 (0) |
| History of cancer | 272 (1) | 243 () |
| Donor CMV positive | 217 (1) | 195 () |
| Donor EBV positive | 12,230 (35) | 11,681 (35) |
| **Recipient** |  |  |
| Age at HTx (years) | 0 (0) | 0 (0) |
| Gender | 0 (0) | 0 (0) |
| Transplant era | 0 (0) | 0 (0) |
| Ischemic time (hours) | 1,189 (3) | 1,128 (3) |
| Etiology heart failure | 2 (0) | 2 (0) |
| CMV serostatus | 2,020 (6) | 1,937 (6) |
| EBV serostatus | 6,076 (17) | 5,816 (17) |
| VAD pre-HTx | 5,390 (15) | 5,141 (15) |
| Malignancies pre-HTx | 240 (1) | 227 (1) |
| Diabetes pre-HTx | 372 (1) | 355 (1) |
| Hypertension pre-HTx | 7,270 (21) | 6,791 (20) |
| Smoking pre-HTx | 8,354 (24) | 7,959 (24) |
| Creatinine pre-HTx (Mg/dL) | 565 (2) | 532 (2) |
| eGFR pre-HTx (mL/min/1.73m^2^) | 727 (2) | 685 (2) |
| Induction therapy | 310 (1) | 294 (1) |
| Discharge CNI | 806 (2) | 758 (2) |
| Acute rejection until discharge | 7,460 (21) | 7,126 (21) |
| Acute rejection discharge and 1 year | 554 (2) | 465 (1) |
| Follow-up duration (years) | 0 (0) | 0 (0) |

Number of missing values on baseline characteristics of the patients included in the study before imputation. All data is given in absolute numbers with percentages.

Abbreviations: CMV, cytomegalovirus; CNI, calcineurin inhibitor; EBV, Epstein Barr virus; eGFR, estimated glomerular filtration rate; HTx, heart transplant; VAD, ventricular assist device

**Supplemental Table S3. Competing risk cumulative incidence of any malignancy, solid-organ malignancy, PTLD, and skin malignancy post-HTx.**

| **Years Post-transplant** | **Any Malignancy (N=34,873)** | | **PTLD (N=** **34,202)** | | **Skin malignancy (N=** **34,265)**  **Skin Cancer (N=** **34,265)** | | **Solid-organ malignancy (N=** **34,852)** | |
| --- | --- | --- | --- | --- | --- | --- | --- | --- |
|  | **Cumulative Incidence** | **95% Confidence Interval** | **Cumulative Incidence** | **95% Confidence Interval** | **Cumulative Incidence** | **95% Confidence Interval** | **Cumulative Incidence** | **95% Confidence Interval** |
| 1 | 4.07% | (3.86%, 4.28%) | 0.52% | (2.05%, 2.36%) | 1.42% | (1.3%, 1.55%) | 2.20% | (2.05%, 2.36%) |
| 2 | 6.77% | (6.51%, 7.04%) | 0.82% | (2.85%, 3.21%) | 3.10% | (2.92%, 3.29%) | 3.03% | (2.85%, 3.21%) |
| 3 | 9.05% | (8.75%, 9.36%) | 1.06% | (3.51%, 3.91%) | 4.61% | (4.38%, 4.84%) | 3.70% | (3.51%, 3.91%) |
| 4 | 11.33% | (10.99%, 11.68%) | 1.35% | (4.11%, 4.56%) | 6.14% | (5.88%, 6.41%) | 4.33% | (4.11%, 4.56%) |
| 5 | 13.17% | (12.79%, 13.55%) | 1.61% | (4.76%, 5.25%) | 7.23% | (6.94%, 7.53%) | 5.00% | (4.76%, 5.25%) |
| 6 | 15.04% | (14.63%, 15.45%) | 1.88% | (5.39%, 5.92%) | 8.46% | (8.14%, 8.79%) | 5.65% | (5.39%, 5.92%) |
| 7 | 16.83% | (16.39%, 17.27%) | 2.07% | (6.03%, 6.59%) | 9.66% | (9.31%, 10.02%) | 6.31% | (6.03%, 6.59%) |
| 8 | 18.36% | (17.89%, 18.83%) | 2.33% | (6.57%, 7.17%) | 10.57% | (10.19%, 10.95%) | 6.87% | (6.57%, 7.17%) |
| 9 | 19.77% | (19.27%, 20.26%) | 2.55% | (7.13%, 7.78%) | 11.47% | (11.08%, 11.88%) | 7.45% | (7.13%, 7.78%) |
| 10 | 21.23% | (20.71%, 21.75%) | 2.75% | (7.74%, 8.43%) | 12.38% | (11.96%, 12.81%) | 8.08% | (7.74%, 8.43%) |
| 11 | 22.58% | (22.03%, 23.14%) | 2.94% | (8.41%, 9.15%) | 13.23% | (12.78%, 13.69%) | 8.78% | (8.41%, 9.15%) |
| 12 | 23.86% | (23.28%, 24.45%) | 3.15% | (8.79%, 9.57%) | 14.07% | (13.59%, 14.55%) | 9.18% | (8.79%, 9.57%) |
| 13 | 24.63% | (24.02%, 25.24%) | 3.23% | (9.32%, 10.16%) | 14.63% | (14.13%, 15.14%) | 9.74% | (9.32%, 10.16%) |
| 14 | 25.70% | (25.05%, 26.34%) | 3.42% | (9.83%, 10.73%) | 15.19% | (14.66%, 15.72%) | 10.28% | (9.83%, 10.73%) |
| 15 | 26.62% | (25.94%, 27.30%) | 3.57% | (10.4%, 11.38%) | 15.75% | (15.19%, 16.32%) | 10.88% | (10.4%, 11.38%) |

In this table the cumulative incidences of all malignancy, solid-organ malignancy, PTLD, and skin malignancy are demonstrated after competing risk analysis with death as competing outcome.

Abbreviations: PTLD, post-transplant lymphoproliferative disease.

**Supplemental Table S4. Categorical risk factors for any malignancy post-HTx (N=33,345)**

| **Risk Factor** | **Comparison** | **Hazard Ratio** | **95% Confidence Interval** | **P-value** |
| --- | --- | --- | --- | --- |
| Recipient CKD stage | Stage 2 (eGFR 60-89) vs Stage 1 (eGFR 90+) | 1.04 | (0.97, 1.11) | 0.26 |
|  | Stage 3A (eGFR 45-59) vs Stage 1 (eGFR 90+) | 1.03 | (0.95, 1.12) | 0.42 |
|  | Stage 3B (eGFR 30-44) vs Stage 1 (eGFR 90+) | 0.97 | (0.87, 1.08) | 0.63 |
|  | Stage 4-5 (eGFR <30) vs Stage 1 (eGFR 90+) | 1.17 | (1.02, 1.35) | 0.023 |
| Recipient gender | Female vs Male | 0.64 | (0.60, 0.69) | <0.001 |
| Recipient diagnosis | ICM vs NICM | 1.15 | (1.09, 1.22) | <0.001 |
|  | Other diagnoses vs NICM | 1.22 | (1.11, 1.34) | <0.001 |
| Recipient pre-HTx diabetes | Yes vs No | 0.90 | (0.85, 0.96) | 0.001 |
| Recipient pre-HTx malignancies | Yes vs No | 1.87 | (1.72, 2.03) | <0.001 |
| Recipient CMV serostatus | Negative vs Positive | 1.23 | (1.17, 1.30) | <0.001 |
| Recipient pre-HTx smoking | Yes vs No | 1.06 | (1.00, 1.13) | 0.051 |
| Recipient pre-HTx VAD | Yes vs No | 0.93 | (0.87, 0.99) | 0.03 |
| Induction type | IL-2RA vs None | 0.98 | (0.92, 1.04) | 0.48 |
|  | Monoclonal vs None | 1.06 | (0.94, 1.21) | 0.34 |
|  | Polyclonal vs None | 1.04 | (0.97, 1.12) | 0.23 |
|  | Multiple Types vs None | 1.29 | (1.06, 1.56) | 0.009 |
| Discharge maintenance immunosuppression | CYA vs TAC | 1.13 | (1.07, 1.20) | <0.001 |
|  | No CNI vs TAC | 0.86 | (0.71, 1.03) | 0.10 |
| Treated acute rejection between discharge and 1 year | Yes vs No | 1.17 | (1.10, 1.25) | <0.001 |

Multivariable Cox regression model demonstrating risk factors for all malignancy post-HTx with hazard ratios and confidence intervals.

Abbreviations: CKD, chronic kidney disease; CMV, cytomegalovirus; CNI, calcineurin inhibitor; CYA, cyclosporine; eGFR, estimated glomerular filtration rate; HTx, heart transplantation; IL-2RA, interleukin-2 receptor antagonist; TAC, tacrolimus; VAD, ventricular assist device.

**Supplemental Table S5. Categorical risk factors for solid-organ malignancy post-HTx (N=33,324)**

| **Risk Factor** | **Comparison** | **Hazard Ratio** | **95% Confidence Interval** | **P-value** |
| --- | --- | --- | --- | --- |
| Recipient CKD stage | Stage 2 (eGFR 60-89) vs Stage 1 (eGFR 90+) | 0.98 | (0.87, 1.09) | 0.65 |
|  | Stage 3A (eGFR 45-59) vs Stage 1 (eGFR 90+) | 1.00 | (0.87, 1.14) | 0.96 |
|  | Stage 3B (eGFR 30-44) vs Stage 1 (eGFR 90+) | 0.92 | (0.77, 1.11) | 0.40 |
|  | Stage 4-5 (eGFR <30) vs Stage 1 (eGFR 90+) | 1.35 | (1.07, 1.69) | 0.01 |
| Recipient gender | Female vs Male | 0.91 | (0.81, 1.02) | 0.10 |
| Recipient diagnosis | ICM vs NICM | 1.20 | (1.09, 1.32) | <0.001 |
|  | Other diagnoses vs NICM | 0.96 | (0.81, 1.14) | 0.62 |
| Recipient pre-HTx malignancies | Yes vs No | 1.64 | (1.43, 1.89) | <0.001 |
| Recipient CMV serostatus | Negative vs Positive | 1.09 | (0.99, 1.19) | 0.07 |
| Recipient pre-HTx smoking | Yes vs No | 1.25 | (1.13, 1.38) | <0.001 |
| Treated acute rejection between discharge and 1 year | Yes vs No | 1.15 | (1.04, 1.28) | 0.009 |
| Transplant era | Jan 2009-Jun 2017 vs Jan 2000-Dec 2008 | 0.68 | (0.61, 0.75) | <0.001 |

Multivariable Cox regression model demonstrating risk factors for solid-organ malignancy post-HT with hazard ratios and confidence intervals.

Abbreviations: CKD, chronic kidney disease; CMV, cytomegalovirus; eGFR, estimated glomerular filtration rate; HTx, heart transplantation ICM, ischemic cardiomyopathy, NICM, non-ischemic cardiomyopathy.

**Supplemental Table S6. Categorical risk factors for PTLD post-HTx (N=32,678)**

| **Risk Factor** | **Comparison** | **Hazard Ratio** | **95% Confidence Interval** | **P-value** |
| --- | --- | --- | --- | --- |
| Recipient CKD stage | Stage 2 (eGFR 60-89) vs Stage 1 (eGFR 90+) | 0.93 | (0.76, 1.15) | 0.52 |
|  | Stage 3A (eGFR 45-59) vs Stage 1 (eGFR 90+) | 0.89 | (0.68, 1.15) | 0.37 |
|  | Stage 3B (eGFR 30-44) vs Stage 1 (eGFR 90+) | 0.79 | (0.57, 1.08) | 0.13 |
|  | Stage 4-5 (eGFR <30) vs Stage 1 (eGFR 90+) | 0.73 | (0.53, 1.01) | 0.057 |
| Recipient gender | Female vs Male | 0.85 | (0.70, 1.02) | 0.08 |
| Recipient diagnosis | ICM vs NICM | 1.30 | (1.02, 1.64) | 0.033 |
|  | Other diagnoses vs NICM | 1.22 | (0.89, 1.68) | 0.21 |
| Recipient pre-HTx malignancies | Yes vs No | 1.95 | (1.45, 2.63) | <0.001 |
| Recipient CMV serostatus | Negative vs Positive | 1.29 | (1.09, 1.54) | 0.004 |
| Recipient EBV serostatus | Negative vs Positive | 1.58 | (1.30, 1.92) | <0.001 |
| Treated acute rejection between discharge and 1 year | Yes vs No | 1.23 | (1.01, 1.49) | 0.036 |
| Donor EBV serostatus | Negative vs Positive | 0.70 | (0.50, 0.97) | 0.034 |

Multivariable Cox regression model demonstrating risk factors for PTLD post-HTx with hazard ratios and confidence intervals.

Abbreviations: CKD, chronic kidney disease; CMV, cytomegalovirus; EBV, Epstein Barr virus; eGFR, estimated glomerular filtration rate; HTx, heart transplantation; ICM, ischemic cardiomyopathy; NICM, non-ischemic cardiomyopathy.

**Supplemental Table S7. Categorical risk factors for skin malignancies post-HTx (N=32, 738)**

| **Risk Factor** | **Comparison** | **Hazard Ratio** | **95% Confidence Interval** | **P-value** |
| --- | --- | --- | --- | --- |
| Recipient CKD stage | Stage 2 (eGFR 60-89) vs Stage 1 (eGFR 90+) | 1.03 | (0.95, 1.12) | 0.48 |
|  | Stage 3A (eGFR 45-59) vs Stage 1 (eGFR 90+) | 1.01 | (0.91, 1.12) | 0.87 |
|  | Stage 3B (eGFR 30-44) vs Stage 1 (eGFR 90+) | 0.91 | (0.79, 1.06) | 0.22 |
|  | Stage 4-5 (eGFR <30) vs Stage 1 (eGFR 90+) | 1.06 | (0.86, 1.31) | 0.59 |
| Recipient gender | Female vs Male | 0.48 | (0.43, 0.54) | <0.001 |
| Recipient diagnosis | ICM vs NICM | 1.11 | (1.03, 1.20) | 0.006 |
|  | Other diagnoses vs NICM | 1.40 | (1.24, 1.58) | <0.001 |
| Recipient pre-HTx diabetes | Yes vs No | 0.83 | (0.77, 0.90) | <0.001 |
| Recipient pre-HTx hypertension | Yes vs No | 0.94 | (0.87, 1.01) | 0.076 |
| Recipient pre-HTx malignancies | Yes vs No | 1.80 | (1.61, 2.01) | <0.001 |
| Recipient CMV serostatus | Negative vs Positive | 1.36 | (1.27, 1.46) | <0.001 |
| Induction type | IL-2RA vs None | 1.02 | (0.94, 1.11) | 0.58 |
|  | Monoclonal vs None | 1.30 | (1.11, 1.52) | 0.001 |
|  | Polyclonal vs None | 1.13 | (1.03, 1.24) | 0.01 |
|  | Multiple Types vs None | 1.13 | (0.85, 1.50) | 0.42 |
| Discharge immunosuppression | CYA vs TAC | 1.22 | (1.12, 1.32) | <0.001 |
|  | No CNI vs TAC | 0.81 | (0.63, 1.04) | 0.11 |
| Treated acute rejection at discharge | Yes vs No | 0.88 | (0.76, 1.02) | 0.10 |
| Treated acute rejection between discharge and 1 year | Yes vs No | 1.18 | (1.08, 1.28) | <0.001 |
| Transplant era | Jan 2009-Jun 2017 vs Jan 2000-Dec 2008 | 1.20 | (1.10, 1.31) | <0.001 |

Multivariable Cox regression model demonstrating risk factors for skin malignancies post-HTx with hazard ratios and confidence intervals.

Abbreviations: CKD, chronic kidney disease; CMV, cytomegalovirus; CYA, cyclosporine; eGFR, estimated glomerular filtration rate; HTx, heart transplantation; ICM, ischemic cardiomyopathy; IL-2RA, interleukin-2 receptor antagonist; NICM, non-ischemic cardiomyopathy; TAC, tacrolimus.
