# Supplementary figures and images for: Influence of chronic kidney disease and other risk factors pre-heart transplantation on malignancy incidence post-heart transplantation
Source: Front Cardiovasc Med. 2023 Apr 3;10:1145996. doi: 10.3389/fcvm.2023.1145996 (PMC10106779; doi:10.3389/fcvm.2023.1145996)

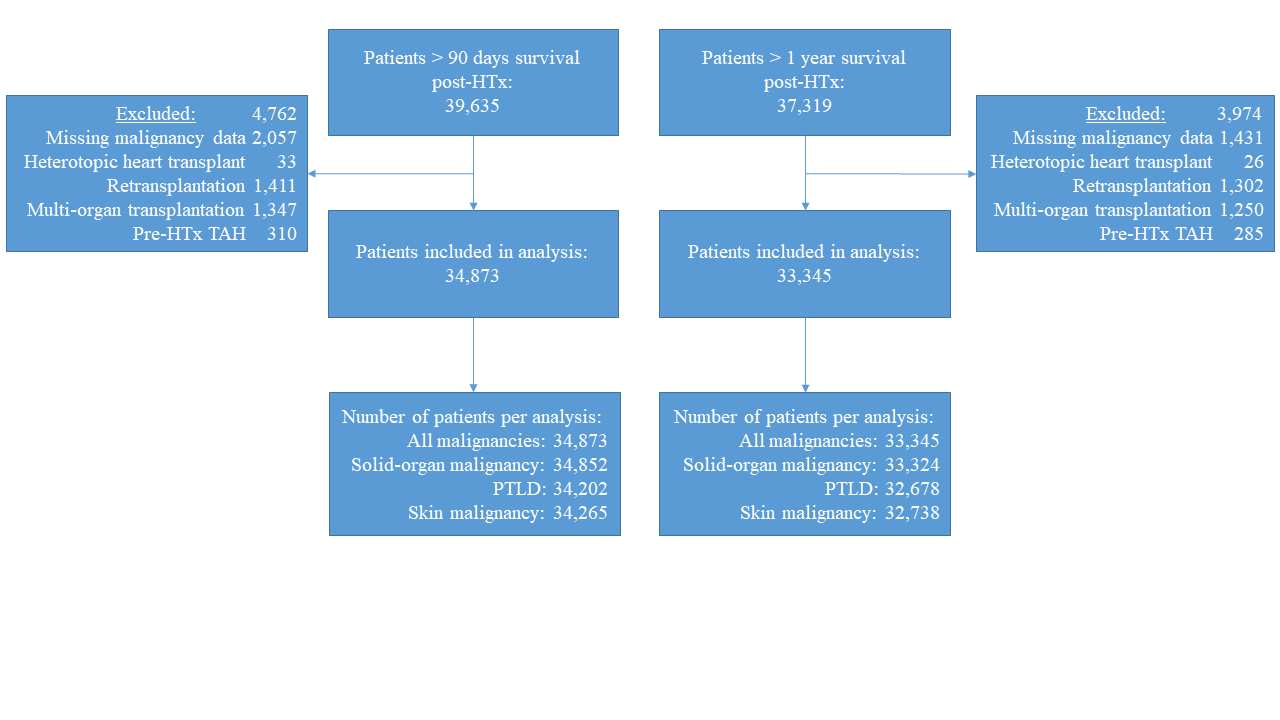

Supplement: Supplementary file 2 [file Image1.tif]

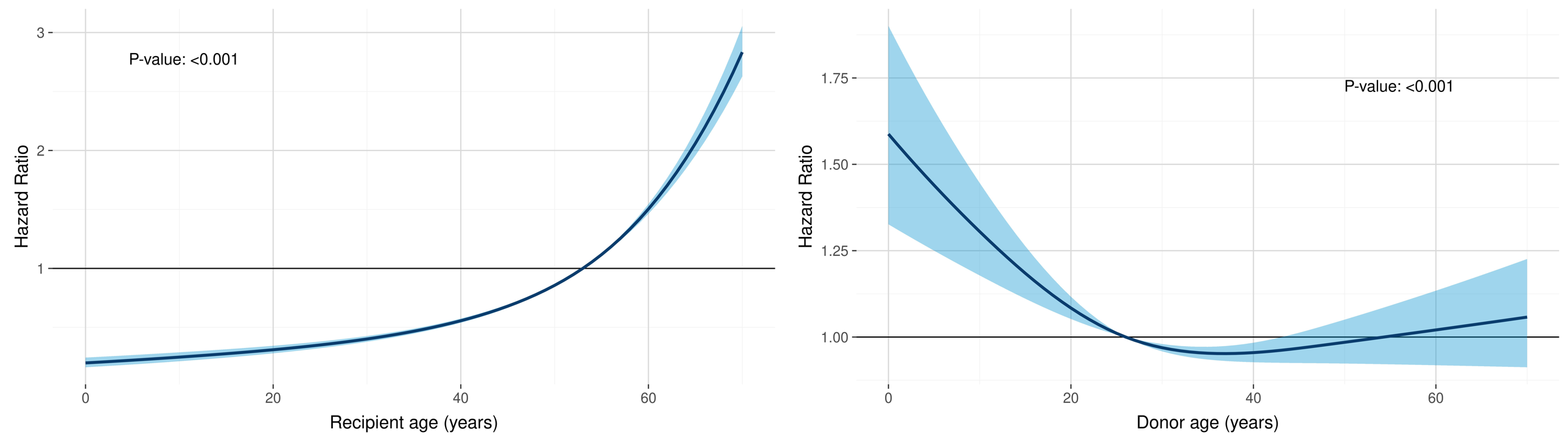

Supplement: Supplementary file 3 [file Image2.jpg]

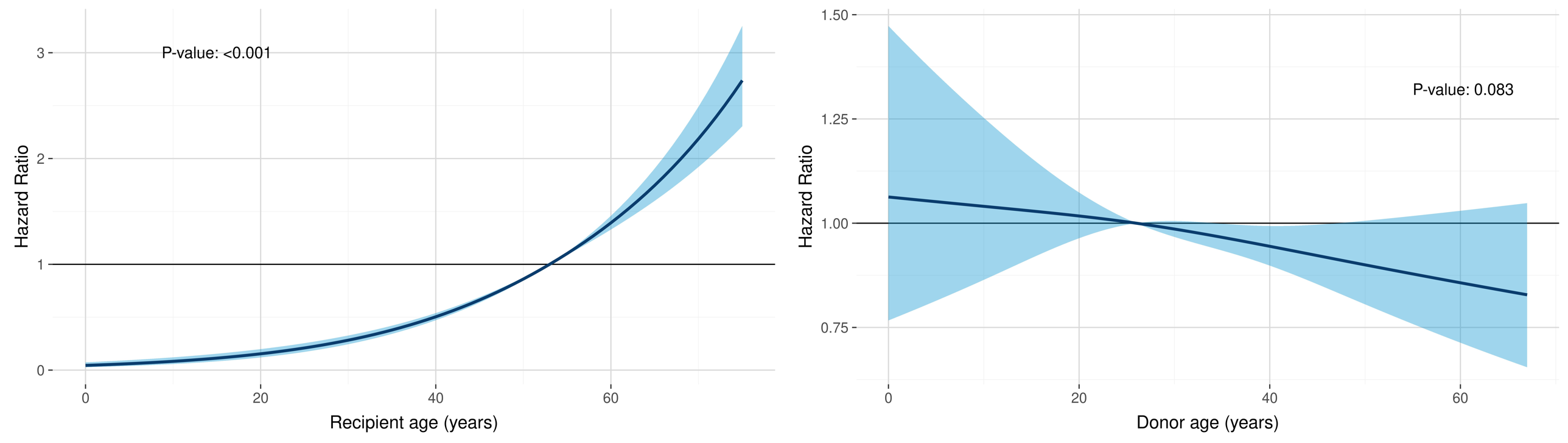

Supplement: Supplementary file 4 [file Image3.jpg]

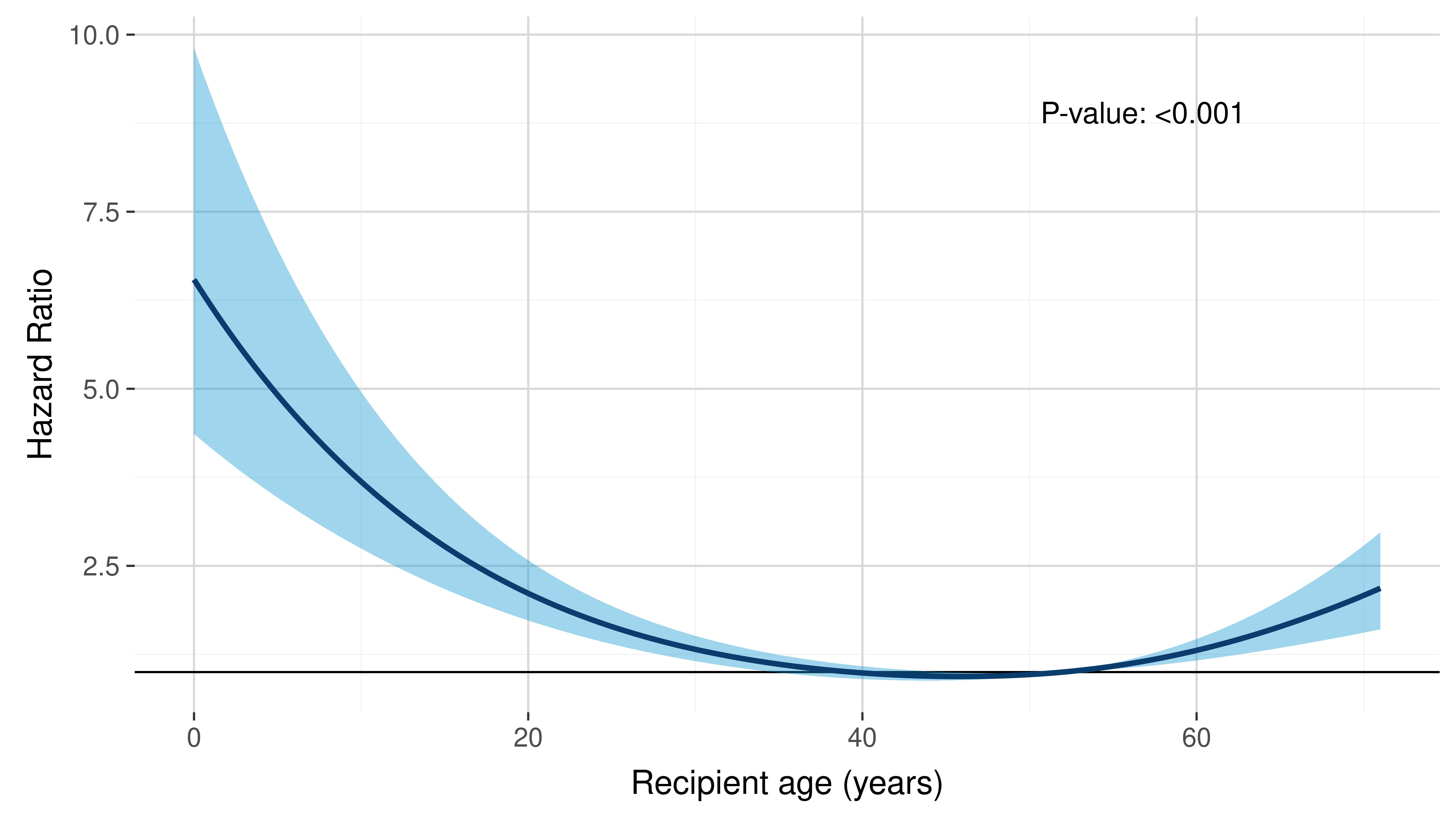

Supplement: Supplementary file 5 [file Image4.jpg]
