# Supplementary material for: Influence of chronic kidney disease and other risk factors pre-heart transplantation on malignancy incidence post-heart transplantation
Source: Front Cardiovasc Med. 2023 Apr 3;10:1145996. doi: 10.3389/fcvm.2023.1145996 (PMC10106779; doi:10.3389/fcvm.2023.1145996)
Supplement: Supplementary file 7 [file Image6.pdf]

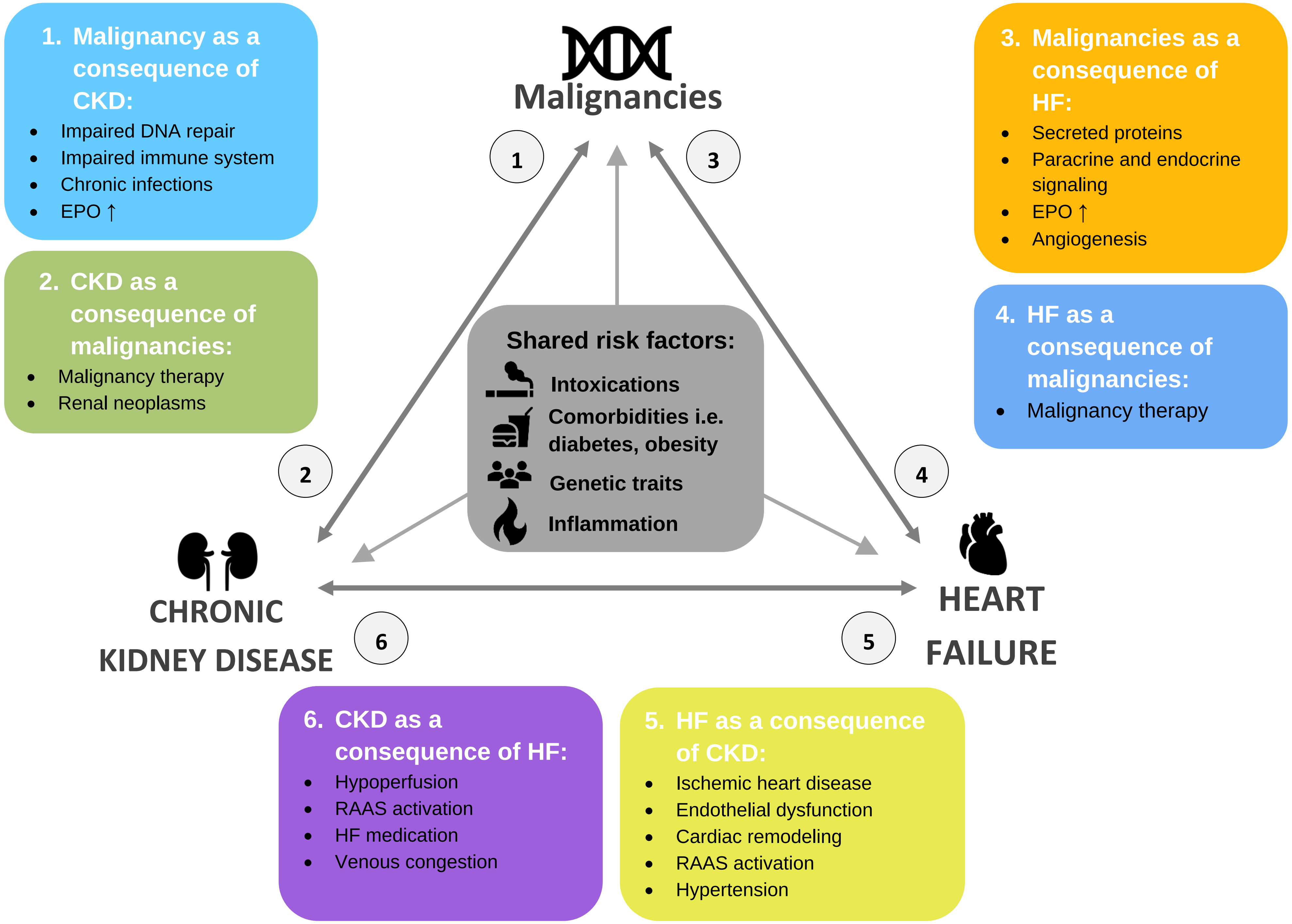

Figure S6. The pathways in which heart failure, chronic kidney disease, and malignancies might be related.

Abbreviations: CKD = chronic kidney disease, DNA = deoxyribonucleic acid, EPO = erythropoietin, HF = heart failure, RAAS = renin-angiotensin-aldosterone system

Reprinted with permission [11]
